# Dental Students’ Perceptions before and after Attending a Centre for Children with Special Needs: A Qualitative Study on Situated Learning

**DOI:** 10.3390/dj8030069

**Published:** 2020-07-03

**Authors:** Hani T. Fadel, Khlood Baghlaf, Afnan Ben Gassem, Hala Bakeer, Alla T. Alsharif, Saba Kassim

**Affiliations:** 1 Department of Preventive Dental Sciences, Taibah University Dental College & Hospital, Prince, Naif Ibn Abdulaziz Road, Al-Madinah Al-Munawwarah 42353, Saudi Arabia; hani.fadel@yahoo.com (H.T.F.); halabakeer80@live.com (H.B.); dr-alsharif@hotmail.com (A.T.A.); saba262003@gmail.com (S.K.); 2 Department of Pediatric Dentistry, Faculty of Dentistry, King Abdulaziz University, Jeddah 80209, Saudi Arabia; 3 Department of Pediatric Dentistry and Orthodontics, Taibah University Dental College & Hospital, Prince, Naif Ibn Abdulazia, Al-Madinah Al-Munawwarah 42353, Saudi Arabia; a_bengassem@hotmail.com

**Keywords:** dental students, special needs children, situated learning, fieldwork

## Abstract

**Objectives:** To develop an in-depth understanding of the perceptions and experiences of senior dental students before and after fieldwork visits to a centre for children with special needs. **Methods:** A qualitative study utilised open-ended questions and involved 39 fifth-year dental students. A thematic analysis of the collected responses was undertaken, and a hierarchy of themes and subthemes were developed. **Results:** Analysis of the pre-visit responses revealed three main themes and a number of subthemes: ‘negative expectations’, ‘positive expectations’, and ‘pain expectations’. Similarly, four main themes with a number of subthemes emerged from the post-visit responses: ‘positive perceptions’, ‘negative perceptions’, ‘oral problems observed’, and ‘recommendations’. **Conclusions:** Within the study limits, different perspectives were extracted. Prior to the fieldwork visit, students expressed their lack of self-confidence and inadequate preparation. Following the situated learning visit experience, students’ perceptions of managing special needs children was positively influenced. Students were happy to be exposed to such an experience, but hoped for better organisation and specialised supervision in the future.

## 1. Introduction

The number of children with special needs in Saudi Arabia (SA) is steadily increasing. Recent data from a national survey found that out of 7,849,953 Saudis under 19 years of age, 209,573 (2.7%) reported having a long-standing condition or disability, accounting for a prevalence rate of 2670 per 100,000 people [1]. Across all administrative regions in Saudi Arabia, Al-Madinah Al-Munawwarah had the highest rates of disabilities related to mobility (57,343) and hearing (17,445) [1].

Through the National Society for Human Rights, Saudi Arabia has historically claimed responsibility of recognising and supporting the rights of people with disabilities, either directly or by enforcing civil rights to ensure their dignified living. Saudi Arabia has also improved services provided to people with disabilities within a framework of legality and equality [2,3]. This disability rights movement has offered appropriate patient-centred dental and medical care, allowing high-risk children (including those with special needs) to obtain priority treatment.

However, dental care access for this segment of society remains challenging due to the lack of general dentists with the necessary expertise, such as behaviour management skills under traditional dental settings [4]. This area of dentistry remains neglected, largely unrecognised and has not received full attention in undergraduate or postgraduate dental education [2]. Some dentists have explicitly reported that patient behaviour, disability, or disease may affect their practice patterns and their willingness to treat children with special needs [5]. Previous studies have shown that general dentists would be more confident when treating patients with special needs if they had been prepared for or exposed to this segment of society during their undergraduate training [6]. Therefore, an urgent demand has been made by researchers for dental curricula to develop undergraduate dental students’ clinical competencies in managing patients with mental and/or physical disabilities in compliance with accepted standards [7]. Treatment of children with special needs is usually touched upon as part of the didactic component of a curriculum, but real-life practical experience is often lacking, hence the importance of the current study.

Student fieldwork visits to children with special needs centres are beneficial for detecting children’s dental problems at an early stage, increasing their family’s oral health awareness, and reducing the risk of preventable oral diseases. Fieldwork visits are often lauded as a crucial component in students’ training, essentially serving as situated learning experiences. The situated learning model was first defined by Brown et al., based on the notion that knowledge is contextually situated and fundamentally influenced by the activity, context, and culture in which it is used [8]. A situated learning experience can allow students to use critical thinking strategies and become aware of complex social and professional issues related to the oral health care of individuals with special needs [9,10,11]. 

Studies on students’ perceptions of fieldwork visits to children with special needs centres are limited, especially within a Saudi Arabian context. There is a lack of knowledge on whether these visits help increase students’ understanding of and abilities to deal with the children. In addition, undergraduate students’ perceptions of learning how to manage children with special needs to be assessed. The aim of this qualitative study was thus to develop an in-depth understanding of the perceptions and experiences of senior dental students before and after fieldwork visits to a centre of children with special needs. 

## 2. Methods

### 2.1. Setting and Sampling

This qualitative study took place at Taibah University Dental College and Hospital, Al-Madinah Al-Munawwarah, Saudi Arabia. A purposive sample of 39 students (male = 17, female = 22) were invited to participate in the study by email. The invitation included a study information sheet and a questionnaire via Google Forms (Google LLC, Mountain View, CA, USA). Inclusion criteria were fifth-year dental students who were scheduled to visit a centre for children with special needs in Madinah as part of their curricular fieldwork practice. Students who did not visit the centre were excluded from the study. The centre manages children with special needs or disabilities such as communication difficulties, learning difficulties, physical disabilities, emotional or mental disorders, behavioural disorders, or intellectual giftedness. During visits, students followed the American Association of Paediatric Dentistry guidelines by promoting positive reinforcement rewards for any desired behaviours, thereby strengthening the likelihood of recurrence of those behaviours. Children were given customised balloons made out of dental rubber gloves to keep them at ease and entertained. In addition, children were given a toothbrush and a toothpaste to reinforce oral hygiene practices.

### 2.2. Study Design, Questions and Ethical Considerations

A phenomenological qualitative design was undertaken, which focused on the lived experience of the students during their visit to a centre of children with special needs. As there were no readily available questionnaires that would address the aim of the research, a topic guide with open-ended questions was adopted to investigate the full range of experiences and perceptions from the target group. The research team, which included two researchers (K.B., A.B.G.) who are experienced in qualitative research, developed the open-ended questionnaires based on relevant literature [7] and the study objectives. The questionnaires were piloted on five dental students, from different dental college in SA, before the start of the study to ensure the clarity and relevance of the questions. The questionnaire used before the fieldwork visit (Table 1) contained eight open-ended questions divided into four sections: expectations about visiting the children with special needs centre, expectations about the oral status of children with special needs, expectations about the special needs children’s dental pain experience, and additional information. 

The questionnaire that was used after the visit (Table 2) contained 12 open-ended questions divided into four sections: the general experiences of the visit, students’ awareness of the children’s oral status, students’ awareness of the children’s dental pain experiences, and additional information. 

The first questionnaire was sent to dental students three days before the fieldwork visit to the children with special needs centre, while the second questionnaire was sent three days after the visit. The participants were encouraged to express their thoughts, views, feelings, actions and experiences and to elaborate on particular areas they regarded as important. They were free to write their perceptions in either Arabic or the English language. All Arabic responses were translated into English to facilitate the analysis. Students were instructed to submit their filled Google Forms after completion. These were then transferred to Excel spreadsheet (Microsoft Excel, 2016 Corp., Redmond, WA, USA) for storage and retrieval for analysis.

Qualitative methods in the form of semi structured questionnaires were used to collect the data. Although semi structured face to face interviews were initially considered as the data collection method, a decision was made to allow the participants to write down their answers and submit them instead, as many of the participants expressed their concern of portraying their perceptions directly to the researcher who is also a faculty member. This allowed for more honest and unbiased responses from the participants. It is well known that semi structured questionnaires are a valid method of data collection that enables the participants to elicit their perceptions and obtain rich and deep information in an insufficiently researched area [12]. Using semi structured questionnaires can help in investigating how people understand and interpret their social surroundings, generating issues and ideas that can facilitate a deeper understanding of the social phenomena. 

### 2.3. Ethical Considerations 

Ethical approval was granted by the Taibah University College of Dentistry Research and Ethics Committee (Reference No: TUCDREC/20191029/Hfadel, 29 October 2019). The participants were informed that all their responses would remain anonymous, that their participation in this part of the outreach activity was completely voluntary, and they were free to withdraw at any time without any impact on their grades. The completion of the questionnaire was considered as a proxy for the informed consent. 

### 2.4. Data Analysis

The participants’ answers were reviewed, and responses for each question were compiled together to facilitate the analysis. The data was analysed thematically following the steps set out by Ritchie et al. [13]. Initially, three transcripts were read twice to allow the researcher to understand the breadth and depth of the data, then an initial thematic framework was developed by grouping all the responses under each topic and rearranging the topics by identifying underlying ideas or ‘themes’ that linked particular items together. Data under each theme were coded and regrouped according to different levels of generality as themes and subthemes. Finally, elements from all the responses were identified as well as the dimensions that differentiated them, yielding a set of categories and higher-order key themes [14,15]. The researcher analysed the data in parallel with the submission of the questionnaires by the participants to make sure data saturation was reached and no new topics were arising.

Multiple coding was performed to investigate inter-rater reliability between the researchers (K.B., A.B.G.) who performed the thematic analysis [16]. Disagreements in coding between the two researchers were discussed by the research team, resulting in a final coding of themes via consensus. Each theme identified in the analysis, together with illustrative quotations from the participants; were presented in the results. Summaries of the quotations from all participants and the context in which they were presented were emailed to the participants to ensure that the interpretations were accurate, and that the conclusions were representative of their lived experiences. The COREQ (Consolidated Criteria for Reporting Qualitative research) checklist was followed for the reporting of this study [17]. 

## 3. Results

Twenty-two participants (10 males and 12 females, response = 56%) returned the questionnaires before the fieldwork visit, while 14 participants (five males and nine females, response = 36%) completed the questionnaires after the fieldwork visit. No new themes and/or subthemes were identified, and therefore, data collection/recruitment was discontinued at this point. 

The analysis revealed a number of themes related to the participants’ perceptions before and after the fieldwork visits. In what follows, each theme is presented with a quotation as an example, followed by the participants’ code.

### 3.1. Students’ Perceptions before the Fieldwork Visit Experience

The analysis revealed three main themes with a number of subthemes under each one related to the participants’ expectations regarding their upcoming fieldwork visit to the children with special needs centre. These themes were ‘negative expectations’, ‘positive expectations’, and ‘pain expectations’ (Table 3). 

#### 3.1.1. Theme 1: Negative Expectations 

Most of the participants were not excited about the fieldwork visit to children with special needs centre. They were not fully prepared to deal with the children because of their lack of clinical training and lack of confidence in managing children with special needs. In addition, some participants expected the visit to be unorganised and felt that the timing was wrong and premature.

*‘The children will be scared and apprehensive. It’ll take a long time to get them to cooperate. The place won’t be organised’.*

*(F7)*

*‘The concept is good, but till now the preparation for the programme is weak. I had no training on how to deal with them’.*

*(M16)*

Several participants expected some noncooperative children that could be difficult to manage either because they have a physical disability preventing them from cooperating, or simply because they are scared and they felt that this will put them in a stressful situation.

*‘I expect to visit people who have a lot of medical problems. I strongly believe that most of them can’t even open their mouths due to their condition’.*

*(M3)*

#### 3.1.2. Theme 2: Positive Expectations 

The participants also shared some positive expectations about the visit. They believed that it would give them the opportunity to learn how to manage children with special needs in addition to the chance to learn about a range of dental and medical problems that these children may face.

*‘Good experience. Maybe the children will learn from us how to clean and take care of their mouths’.*

*(M17)*

*‘Well, it will be a good experience as you can interact, diagnose, and examine the special needs children and it’s really the first time I interact with them’.*

*(M4)*

Other positive expectations that were expressed included the privilege of serving the community and helping to increase awareness of the special needs children and their families about oral health issues.

*‘This is a new experience and exposure for a different kind of examination. Also, this can be good community work’.*

*(M16)*

*‘The advantages are to help the community by teaching them about maintaining their oral health and physical health as a whole’.*

*(M8)*

Finally, the participants also believed that this type of experience may have some religious benefits for them as Muslims by helping them gain Ajar (spiritual rewards from Allah) for examining those children.

*‘The advantages of fieldwork visits are new experiences, exposure to a different kind of examination, doing something good for the community, and earning “Ajar” Insha’Allah’.*

*(M16)*

#### 3.1.3. Theme 3: Pain Expectations in Children with Special Needs 

When the participants were asked about their expectations of the children’s pain experience, the majority replied ‘I don’t know’, while some students expected that children with special needs mostly had severe dental pain but lacked the ability to express it because of their disabilities.

*‘I think that children with special needs suffer from severe dental pain. That’s really sad and they cannot explain it to their dentist or family members’.*

*(M2)*

### 3.2. Students’ Perceptions after the Fieldwork Visit Experience

Analysis of the data revealed four main themes with a number of subthemes under each one related to the participants’ experiences after the fieldwork visit to the special needs centre. These were grouped into ‘positive perceptions’, ‘negative perceptions’, ‘oral problems observed’, and ‘recommendations’ (Table 4).

#### 3.2.1. Theme 1: Positive Perceptions 

Most students were satisfied and had positive perceptions after the visit, as they recognised the importance of being active in the community and helped raise dental awareness among children and their families. In addition, the participants had the opportunity to engage in practice-based learning where they were able to perform dental examinations on patients who were otherwise uncooperative through implementing different techniques and talking to their parents regarding oral hygiene measures.

*‘I think the tooth brushing demonstration for both parents and children was helpful for them. Family should be involved in oral health practice because they are the only ones who take care of the child and seek dental treatment’.*

*(F5)*

Some students also mentioned that they used behaviour management methods that they had learned in theory on uncooperative children, and were able to apply these in real life through tell, show, and do (TSD) as well as live modelling methods.

*‘I put the diagnostic kit on their hands and skin to ensure them nothing is invasive. And we showed them their friends who were cooperative’.*

*(F5)*

*‘Make it simple and easy. Speak kindly to the child to notify him about what you will do before starting examination, which is known as the tell, show, and do technique and avoid instruments, which may make the child less cooperative, like using the explorer, especially those who have mental disabilities’.*

*(M6)*

*‘For uncooperative children, let them watch their friend being examined’.*

*(M3)*

Finally, the students were also exposed to and had the opportunity to engage with patients suffering from various syndromes and disabilities that they would otherwise not necessarily see in normal dental clinical settings, such as downs syndrome, attention deficit hyperactivity disorder (ADHD), physical disabilities, and various mental disabilities.

*‘According to my previous visit, I saw only one child in pain who was uncooperative. He had attention deficit hyperactivity disorder’.*

*(F2)*

*‘The experience was beautiful and useful because it made us deal with new type of patients that we are not used to dealing with in the clinics’.*

*(F12)*

#### 3.2.2. Theme 2: Negative Perceptions 

The participants also expressed negative feelings following their experience for several reasons. Some believed that the programme was inappropriately timed and not well organised, leaving some students with no time to perform dental examinations on the patients:

*‘Well, I did not like the fieldwork b/c it wasn’t well organised. I was an assistant first and did not have time to examine the children myself’.*

*(M1)*

The participants also felt that they were not prepared to face the situation, especially because of the lack of previous training on how to manage children with special needs. Some students also mentioned the lack of supervisors experience, which made the visit even more difficult for them.

*‘The fieldwork visit should be modified. Sorry for saying that but even the supervisors were not qualified enough to deal with some of the patients.’*

*(M1)*

*‘I don’t think I was well prepared to deal with special needs, so I don’t think it was very useful’.*

*(F5)*

*‘No, it was not helpful because it came without any planning, or preparation and training to deal with this group of people’.*

*(F7)*

The setting was considered inappropriate by a few participants as it was done at the centre, which was lacking suitable facilities for dental examinations. For example, a couple of participants mentioned that they used wooden chairs to examine the children, which caused backaches.

*‘Very much expected. I had backache for the whole day after working because of the chairs. But the kids and their guardians were very nice’.*

*(F5)*

Finally, the participants’ negative feelings were related to the patients themselves, as some mentioned that dental management of very young, medically compromised children is something difficult for them. Some students felt that the fieldwork visit was exhausting as some uncooperative children refused to open their mouths, pushed, and bit them during the dental exam.

*‘As male students working with children who are younger than six years, it was so difficult to deal with them. I believe we didn’t get the chance our female colleagues in the department did to learn. They examined children who were older than ten so I believe it’s easier to deal with them and learn more about their needs!’*

*(M2)*

*‘Some children were crying, and they pushed my colleagues during the dental examination and also bit their fingers’.*

*(F6)*

#### 3.2.3. Theme 3: Oral Problems Observed

The participants especially expressed the importance of these fieldwork visits because they did not frequently see these patients at the dental clinic. They were able to identify a variety of oral health problems in most of the patients, who suffered from poor oral hygiene and high plaque levels due to brushing difficulties, periodontal diseases, dental caries, and bruxism.

*‘Poor oral hygiene. They have plaque in almost all teeth and I expected this because they can’t brush well or maintain oral hygiene’.*

*(M10)*

*‘Some children had the habit of bruxism due to amphetamine treatments’. *

*(F4)*

As for dental pain, although many of the students were not able to predict the patients’ experiences before the visit, many were able to describe it after the visit, and they experienced the difficulties of examining this group of children compared to normal children. While some participants found that the patients’ pain was severe, others found it to be less so. Most of the participants also mentioned that the children tended to jump just from having their teeth touched and started to cry from the pain. Generally, they realised the special care that this group of patients required.

*‘The pain was severe. One child started crying when only touching the tooth with a mirror’.*

*(M3)*

*‘I think most children suffered from severe dental pain, but some of this was moderate. Yes, it varies from normal children’.*

*(F2)*

*‘I think the dental pain was severe, but for some of the children it was moderate. It varies from normal children, because as I was measuring the gingival bleeding index, the child felt pain from just touching the gingiva’.*

*(M10)*

#### 3.2.4. Recommendations for Future Fieldwork Visits 

The participants expressed a variety of recommendations that they believed would enhance both the students’ and the patients’ experiences in similar fieldwork visits in the future. All the participants strongly believed that students should be prepared and trained to examine children with special needs before being sent to the field, as this could play a big role in enhancing their confidence and reducing their stress levels.

*‘Training the student before dealing with children with special needs, because it is very stressful to go there without good training’.*

*(M1)*

Some participants expressed the need to visit more rehabilitation centres that serve different groups of the community to enhance their patient management skills.

*‘I hope we can go to more rehabilitation centres; we always see normal children in the clinic’.*

*(F6)*

*‘I think we need to visit more centres for children with special needs’. *

*(F12)*

Some participants recommended the company of more supervisors, in addition to the presence of an experienced dentist who is specialised in treating medically compromised children so they could support the students better and manage the programme more efficiently.

*‘We need more fieldwork visits. It was so helpful for us. However, in the future I would recommend more dentists specialised in treating disabled children to support undergraduate students during dental examination and behaviour management’.*

*(M2)*

Changing the setting was considered necessary by a couple of participants to improve the experience. They suggested bringing the children to the dental college and performing the dental examination in the dental clinic, which would be more comfortable for the students and the patients in addition to facilitating behaviour management.

*‘I believe it’s better to bring them to our clinics for examinations. It was so difficult to examine them on unadjustable wooden chair!!’*

*(M1)*

*‘Most of them are difficult to deal with outside the special clinic’.*

*(F7)*

A few of the participants recommended enhancing the services as they believed that dental examinations alone are not enough. They recommended applying topical fluoride preventive and fissure sealants.

*‘And I think we need to focus on their families to teach them about brushing techniques and fluoride application and fissure sealant, rather than screening the children without actual advantages for them’.*

*(F5)*

Others mentioned that it was stressful for the children to see them in their uniforms and lab coats, which made them anxious during the examinations and visits as a whole. They believed that removing the lab coats would create a less stressful and friendlier environment.

*‘I would recommend in future we do not wear lab coats. I tried that myself and it was faster with less effort’.*

*(F2)*

Finally, many of the participants also believed that the programme needs to be well organised in ways that would accelerate the time needed to complete the dental examinations and, at the same time, allow all the students to fully participate.

*‘Also, I would recommend that examination be done class by class so the students can focus on the children. It wasn’t organised for me the last time’.*

*(F5)*

## 4. Discussion

To the best of our knowledge this was the first study that attempted to understand the experiences of dental students before and after fieldwork visits to a centre for children with special needs in SA. 

### 4.1. Students’ Perceptions before the Fieldwork Visit 

Before the fieldwork visit, the participants expressed a range of positive and negative expectations. They believed that, although the visit would provide them with a new learning experience that would ultimately enhance their knowledge and patient management skills and the opportunity to serve the community, they also believed that they were not ready for the visit. This was due to their lack of preparedness to deal with this group of patients who they expected to be uncooperative and difficult to manage, as well as the lack of preparation of the programme itself. This agrees with a study that explored the assumptions dental students make prior to beginning community-based clinical activities [18]. They found that students looked forward to improving their clinical and patient management skills and knowledge. Persuni et al. reported that negative feelings were expressed by first- and third-year students, who also believed they were not ready to manage special needs patients prior to their rotations and rated highly on their concern of injury to self or patient as well as patient communication and cooperation [19]. It is imperative that students in dentistry and healthcare professions programmes generally put into practice and apply what they have learned in lectures [20,21]. However, the current findings stress the importance of preparing the students both theoretically and practically before exposing them to the real-life situation in the form of fieldwork visits. This may be achieved by delaying such visits until students reach their fifth or sixth year of the dentistry programme, where they would be better trained clinically and have been exposed to paediatric patients, thereby increasing their confidence in engaging with different groups of patients. 

### 4.2. Student’s Perceptions after the Fieldwork Visit 

Participant responses revealed four themes related to the experiences after the fieldwork visit including ‘positive perceptions’, ‘negative perceptions’, ‘oral problems observed’, and ‘recommendations. The participants were happy that they were able to serve the community, experience practice-based learning and the privilege of being exposed to children suffering from various syndromes and disabilities. The participants were also able to express, based on what they saw during the visit, a range of oral problems related to oral hygiene, dental and orthodontic problems, and dental pain that these patients usually suffer from. Some negative expectations that were held before the visit turned into positive experiences after the visit. The fear of managing children with special needs before the visit, for example, turned into feelings of happiness for being able to use behaviour management techniques after the visit. This finding agrees with that in the study of Perusini et al. who found that dental students’ expectations of treating persons with disabilities were generally more negative in nature compared with their experiences after actually being exposed to the patients [19]. 

Other negative experiences that were portrayed before the visit were also reinforced after the visit but with more specificity. Participants were able to pinpoint the various weaknesses that were evident during the activity, which helped them recommend solutions to such problems in order to improve the patients’ and their own experiences. 

### 4.3. Recommendations Articulated by Participants 

One of the main recommendations articulated by the students was their need to be formally trained on managing children with special needs, as well as visiting more rehabilitation centres that serve different groups of the community. Studies have shown that academic dental institutions have a history of underpreparing students to deal with the increasing population of individuals with special needs [22]. Previous studies have suggested that dentists who had been exposed to lectures and hands-on training about children with special needs were more likely to treat those patients with confidence [23]. 

Other recommendations that were suggested by the participants were related to the supervisors, believing that it would be beneficial to increase the number of supervisors who are specialised or experienced in managing medically compromised children. This is a commonly observed finding, where studies in the United States have shown that dental faculty members responsible for teaching students to treat this population are not themselves routinely seeing such patients [24]. Consequently, the objective of having students become competent in managing those patients during their undergraduate training is neither achievable nor realistic. This highlights the importance of training faculty members on managing these patients and/or recruiting dentists who are specialised in the area.

Recommendations related to the services provided during the visit were also proposed by the participants. These included better organisation of the visits to allow all students to participate, schedule the patients to be seen at the dental college for enhanced comfort and performance, and improving the services by adding preventive measures such as the application of topical fluoride and fissure sealants. Although such recommendations may be valid, ideal training circumstances may not always be obtainable. Thus, live fieldwork visits aim to familiarise students on how to handle different scenarios, including working in sub-optimal settings. Therefore, it is important to give students as much detail and advice as possible in advance to help them adjust more flexibly with the situation ahead. For example, limited examination time or minimal patient numbers can be compensated for by students working in pairs or teams, where roles can be distributed among them and the whole team benefits from the experience in its entirety. In addition, the absence of full sets of equipment or instruments would train students to use available resources effectively. A small-group orientation session before students go on their rotations has been found to be an effective way to discuss such details [25].

One final observation by the students was the stressful impact of formal healthcare providers’ clothing and the white lab coats on the children. Interestingly, the ‘white coat effect’ has been reported in the literature and studies have shown that a clinician’s appearance is one of the factors that play a role in nonverbal communication within the child–physician relationship, and that casual or colourful and decorated attire is preferred over the traditional white coat in reducing the apprehension and stress levels of the patients [26]. 

Findings of this study suggest that situated learning in the form of fieldwork visits is a valuable theoretical approach to adopt in dental education programmes, as it aids in the students’ understanding, self-reflection and critical thinking by immersing them in the situation. Theoretically, learning requires social interaction and involvement in a ‘community of practice’ [27]. Lave and Wenger proposed this theory by hypothesising that ‘everyday’ unconscious learning occurs by reference to activity, context and the culture in which it takes place [28]. Therefore, dental schools must empower their students to learn in ways which reduce their dependence on formal teaching and increase their ability to learn from experience and reflective investigation [27].

### 4.4. Limitations of the Study

This study included only a few participants from one dental school, which may limit the breadth and applicability of the findings. Nevertheless, this dental school adopts a traditional curriculum that is typical for most dental schools in Saudi Arabia, and the student population is similar to those of other schools in the Kingdom. Most of the research team were academics affiliated with Taibah University Dental College and Hospital. Therefore, pre-existing relationships or reputations may have influenced (positively or negatively) students’ agreement to participate in the study or their responses afterwards. However, participants were not aware of the goals of the study and were assured anonymity of their responses, without any negative repercussions. Several methods were used to establish the credibility of the study [29], such as multiple coders to establish inter-rater reliability. The depth of exploration meant that the emerging themes were consistent across all comparable responses, suggesting theoretical saturation. Despite these findings, a limitation of this study was the subconscious biases that could have resulted from the researcher influencing the interpretations. Researchers A.B.G. and K.B. are assistant professors in the paediatric and orthodontic department, and this might increase the potential for bias and presumptions when analysing findings from this study. Therefore, to further enhance the validity of the results [30], the researchers discussed the research process and findings with another colleague, who was experienced in qualitative methods to overcome bias and presumptions. Similar challenges are often encountered by novice researchers [31,32].

## 5. Conclusions

Within the limitations of this study, it can be concluded that different perspectives were extracted from dental students before and after fieldwork visits to a centre of children with special needs. Prior to the visit, the students expressed their lack of self-confidence and inadequate preparation. However, the situated learning experience positively influenced the students’ perception of managing children with special needs as shown by their post-visit responses. They were happy to be exposed to rare conditions and syndromes and privileged to serve this special group of the community. Lack of previous training and appropriate facilities were the main reasons why some students perceived the fieldwork visit negatively. Changing the location of the examinations from being onsite into a clinical setting, visiting more rehabilitation centres, and including specialised supervisors were proposed by the dental students to improve the experiences of both the students and the patients. This study highlights the advantages of situated learning activities on dental students’ education and training, as situated learning emphasised students’ confidence, perception and the fieldwork visit experience as a whole.

## Figures and Tables

**Table 1:** The list of the open-ended questions before the fieldwork visit.

**Section 1: The General Expectations of the Visit** 1. What are your expectations of visiting the centre for the children with special needs to screen their oral health? (e.g., expectations could concern the place, the people you meet, etc.) 2. How do you perceive this outreach activity experience as part of your learning as a dental student? 3. Do you feel prepared for this outreach activity? If yes how did you prepare? If no, why aren’t you prepared? 4. What do you think are the advantages and disadvantages of this visit?
**Section 2: Oral Health Status of Children with Special Needs** 5. What experiences, if any, do you have with children with special needs (physical, mental, both)? (If YES, please explain this experience. Otherwise, please write NO) 6. What level of oral health do you expect to find among children with special needs?
**Section 3: Pain Experience of Children with Special Needs** 7. What level and types of pain do you expect to find among children with special needs?
**Section 4: Additional Information** 8. Please add any additional comments regarding your perceptions and expectations of visiting centre for children with special needs centre.

**Table 2:** The list of the open-ended questions after the fieldwork visit.

**Section 1: General Experiences of This Visit** 1. What was your first impression on your visit to the centre of children with special needs? 2. Do you believe this experience was helpful to your dental education? Why? In what way? 3. What oral/dental conditions and oral health variations did you find while screening children with special needs? How did this relate to your expectations before the visit? 4. Rate your experience of working with the children with special needs. Did you find any unexpected conditions? How was this similar or different from your previous experiences? 5. Describe what happened during the oral screening. Were the children with special needs cooperative? Were you able to examine them? Please provide examples. 6. Based on your observations, what approach or approaches would you propose to enhance the process of screening children with special needs, requiring less time and effort?
**Section 2: Oral Health Status of Children with Special Needs** 7. In your opinion, what is the most pressing dental problem in children with special needs? 8. Do you think children’s oral status is related to the diagnosis or severity of their medical problems? If yes, please write an example of any child with special needs you have seen. 9. How would you describe the oral hygiene of children with special needs? Please provide examples from this visit.
**Section 3: Pain Experience of Children with Special Needs** 10. Based on your experience during this visit, how would you describe the dental pain in children with special needs?
**Section 4: Additional Information** 11. Please add any additional comments regarding your experience of visiting the children with special needs centre.

**Table 3:** Coding tree of student perceptions before the fieldwork visit showing the three main themes, their subthemes (bold), and elements (bullet points).

Negative Expectations	Positive Expectations	Pain Expectations
○ **Lack of preparation** ○ Lack of clinical training ○ Lack of confidence ○ Facility not organised ○ Wrong timing ○ **Uncooperative children** ○ Difficult to manage ○ Cannot open mouth ○ Scared ○ Stressful	○ **New learning experience** ○ How to manage ○ Dental problems ○ Medical problems ○ **Increase awareness** ○ Children ○ Parents ○ **Serving the community** ○ **Religious benefits** ○ Ajar	○ Does not know ○ Severe dental pain ○ Inability to communicate

**Table 4:** Coding tree of student perceptions after the fieldwork visit showing the three main themes, their subthemes (bold) and elements (bullet points).

Positive Perceptions	Negative Perceptions	Oral Problems Observed
**Community service** ○ Raising dental awareness **Practice-based learning** ○ Manage uncooperative children ▪ Tell show and do Live modelling Avoid sharp instruments ○ Dental examination ○ Oral health instructions to children and parents **Exposed to children with various syndromes and disabilities** ○ Down Syndrome ○ Attention deficient hyperactivity syndrome ○ Physical disabilities ○ Mental disabilities	**Programme** ○ Inappropriate timing ○ Lack of time ▪ Did not have the chance to examine **Preparation** ○ Lack of training ○ Inexperienced supervisors **Setting** ○ Inappropriate location ○ Lack of facilities ▪ Wooden chair for exam Back ache **Patients** ○ Very young ○ Uncooperative ▪ Refused to open mouth Biting Pushed students away	**Oral health** ○ High plaque levels ○ Difficulty brushing ○ Periodontal problems ○ Dental caries **Dental** ○ Bruxism ○ Side effects of drug use e.g., Amphetamine treatment ○ Crowding **Dental pain** ○ Different than regular patients ○ Severe pain ○ Moderate pain ○ Crying
**Recommendations**		
**Students** ○ Training course before the visit ○ Timing of visit; later in the bachelors’ programme ○ More fieldwork visits **Supervisors** ○ Better qualified ○ Bigger number **Services** ○ Changing the setting ▪ Dental exam in dental clinic ○ Enhanced services ▪ Topical fluoride Fissure sealant ○ Appearance ▪ Remove lab coat ○ Better organisation		
